# Expression and clinical significance of ECHS1 in gastric cancer

**DOI:** 10.7150/jca.88604

**Published:** 2024-01-01

**Authors:** Ting Lu, Liang Sun, Qingmin Fan, Junchen Yan, Dan Zhao, Chunfang Xu, Fenglin Dong

**Affiliations:** 1 Department of Ultrasound, the First Affiliated Hospital of Soochow University, Suzhou 215006, China.; 2 Department of General Surgery, the First Affiliated Hospital of Soochow University, Suzhou 215006, China.; 3 Department of Gastroenterology, the First Affiliated Hospital of Soochow University, Suzhou 215006, China.

## Abstract

**Background:** Gastric cancer (GC), as one of the most common malignant tumors and the 3rd primary cause of death by cancer globally, poses a great threat to public health. Despite many advancements have been achieved in current treatment avenues for GC, the 5-year survival rates of GC patients remain substandard. Short-chain enoyl-CoA hydratase (ECHS1) exerts pro- or anti-cancer activities in different cancer backgrounds. However, its clinical significance and biological role in GC remain vague and need further investigation.

**Methods:** The expression of ECHS1 in GC tumors and adjacent normal tissues was examined using the GEPIA platform and clinical samples. The effects of ECHS1 on GC cell proliferation and migration were evaluated using colony formation and transwell migration assays.

**Results:** ECHS1 was upregulated in GC tumor tissues in both mRNA and protein levels and increased ECHS1 was markedly linked with tumor location, depth of tumor invasion, lymph node metastasis (LNM), and tumor-node-metastasis (TNM) stage of GC patients. High ECHS1 expression was also linked with a shorter overal survival (OS), first progression (FP) and post progression survival (PPS). Further subgroup analysis showed that OS was significantly shorter in GC patients with high ECHS1 expression compared to those with low ECHS1 expression belonging to tumors with T3 stage, N2 stage or in instestinal Lauren subgroup. In addition, cytological experiments showed that there was higher ECHS1 expression in GC cell lines compared to the normal gastric epithelium (GES-1) cells, and ECHS1 can promote GC cell proliferation and migration in vitro.

**Conclusion:** ECHS1 plays an oncogenic role in GC and might be a promising therapeutic target for GC.

## Introduction

Among the most frequent malignant tumors is gastric cancer (GC), which is the 3^rd^ primary cause of death by cancer globally, after lung and colorectal cancers 1, 2. The literature suggests that liver and peritoneal metastases are the primary reason for increased mortality in advanced GC patients 3. Despite multimodality therapy and advancements in surgical protocols, systemic chemotherapy, targeted treatments, and immunotherapies, the 5-year survival rates for GC tumors of stages IA and IB remains low (between 60% and 80%), and the average survival of stage III patients is between 18% and 50% 4. Therefore, a comprehensive analysis of GC pathogenesis, its metastasis mechanism, and the identification of novel tumor indices are significantly required to improve the survival and prognosis of GC patients.

Lipids are crucial cellular components and sources of energy for living organisms. To meet the needs of rapid proliferation, tumor cells undergo important changes in lipid metabolism 5. Excessive lipogenesis and lipid composition alterations are essential for tumor cells 6. Currently, much research on the correlating lipid metabolism and tumors has been carried out, including those focused on acetyl-CoA carboxylase and fatty acid synthase (FASN) 6. Inhibition of these targets has substantial in *vivo* and in *vitro* anti-tumor effects; however, these targeted drugs have indicated side effects, such as weight loss and some toxic reactions. Therefore, the development of novel tumor metabolism-related therapeutic targets is critical.

Short-chain enoyl-CoA hydratase (ECHS1) is the critical enzyme in the 2^nd^ step of fatty-acid β oxidation (FAO) hydration. Mitochondrial FAO is a significant metabolic pathway that provides energy, especially under physiological conditions when glucose is reduced to provide energy as a primary source. In addition to participating in lipid metabolism, ECHS1 is also crucial for various tumor development. In colorectal cancer 7, 8, hepatocellular carcinoma 9, 10, and breast cancer 11, the expression of ECHS1 is markedly elevated than the adjacent healthy tissues and can promote tumor cell growth and migration and chemotherapy resistance. However, in renal cell carcinoma 12, 13, the ECHS1 expression is significantly alleviated than in adjacent tissues and can inhibit tumor cell proliferation. Additionally, colorectal and kidney cancers 8, 13 studies have revealed that ECHS1 expression can be utilized as a molecular index for early diagnosis and prognosis. These research studies indicate that ECHS1 is essentially linked with the development of tumors, but its expression is characterized by tumor heterogeneity. Furthermore, ECHS1 has a potential clinical application prospect in diagnosing and prognosis of malignant tumors.

Currently, the association of ECHS1 with GC is rarely reported. Zhu et al. 14 found that ECHS1 knockdown (KD) could suppress GC cell proliferation and migration. Furthermore, it has been observed that the expression of ECHS1 is enhanced in GC tissues 15; therefore, it could be utilized as a novel marker to assess prognosis. This investigation aimed to further elucidate ECHS1 expression in GC tissues and its association with the GC clinicopathological parameters. Moreover, the clinical prognostic significance of ECHS1 in GC was comprehensively evaluated.

## Methods

### Human GC tissues

Clinical data were acquired from the Gene Expressing Profiling Interactive Analysis (GEPIA; http://gepia.cancer-pku.cn/) platform. The correlation of ECHS1 mRNA expression with GC patient survival, including first progression (FP), overall survival (OS), and post-progression survival (PPS), was assessed via the Kaplan-Meier (K-M) Plotter (https://kmplot.com) database. 77 GC and adjacent healthy gastric tissue samples were acquired from the histopathologically diagnosed GC patients who underwent radical surgery at the Department of General Surgery, the First Affiliated Hospital of Soochow University. No participant received preoperative radio- or chemotherapy. The ethical board of the concerned institute authorized this investigation, and all the participants were first informed about the research, and then their consent was acquired.

### Immunohistochemistry (IHC)

Briefly, the paraffin-submerged tissues were sliced (5 μm thick), tagged at 4℃ with the monoclonal human ECHS1 antibody (dilution 1:100; #11305-1-AP, Proteintech) overnight, stained via staining kit (Zhongshan Biotechnology, Beijing, China), visualized, and then the staining score was assessed based on color intensity and positive cell rate, per previous protocol 16, 17. An average intensity score of ≥4 indicated high expression, and <4 depicted no or low expression.

### Cell lines

Human GC (AGS, MKN45, and BGC823) and healthy gastric epithelium (GES-1) cell lineages were provided by the Shanghai Institute of Biochemistry and Cell Biology, Chinese Academy of Sciences (Shanghai, China) and propagated at 37℃ in Roswell Park Memorial Institute (RPMI) media (Hyclone) augmented with 100 units/ml penicillin G sodium, 10% Fetal Bovine Serum (FBS; Gibco), and 100 μg/ml streptomycin sulfate (Gibco) in 5% CO_2_.

### Construction of Stable Cell Lines

Plasmids that encode human ECHS1 and ECHS1-specific short hairpin RNA (shRNA) (sequence 5'-GCCCATATCGTTTCATAGCTT-3') were utilized for stable overexpression (OE) and KD via lentivirus transduction (GeneChem, Shanghai, China), per the manufacturer's guide. The negative control shRNA sequence is 5'‑TTCTCCGAACGTGTCACGTTT‑3'.

### Protein isolation and Western blot analysis

The protocol applied in our previous research was utilized for this assay 16, 17. Briefly, the whole cellular protein was acquired by incubating cells in ice-cold RIPA lysis buffer (Beyotime Inc, NanTong, China) augmented with protease and phosphatase inhibitors cocktails (Sigma), per the manufacturer's protocol. The acquired proteins were isolated by SDS-PAGE, transplanted on the PVDF membranes, occluded for 1 h using 5% non-fat milk, rinsed, tagged at 4℃ with the indicated primary antibodies with gentle overnight shaking, and then labeled with horseradish peroxidase (HRP)-coupled secondary antibodies. The immunoreactive bands were visualized via enhanced chemiluminescence. The ECHS1 (1:100; #11305-1-AP) and β-actin (1:1000; #66009-1-Ig) antibodies were acquired from Proteintech and utilized in this investigation.

### Colony formation assay

The method from previous research was carried out for this assay 16, 17. Briefly, cells (1000/well) were propagated in six-well plates for 10 days, then the colonies were preserved and stained with the help of 0.1% crystal violet (Beyotime Institute of Biotechnology). Colonies of > 50 cells were quantified via a light microscope (Olympus, Tokyo, Japan).

### Cell migration assay

The method of previous research was followed 16, 17. The migration ability of GC cells was elucidated with the help of Transwell chambers (8.0 µm pore size, Corning Inc., New York, USA). Migrated cells were stained for 15 min using 0.1% crystal violet at ambient temperature.

### Statistical analysis

All the statistical values are depicted as mean ± S.E.M of 3 experimental replicates. A statistically substantial difference was assessed via Student's *t*-test (unpaired or paired, two-tailed) or chi-square test. For survival analysis, K-M and Cox analyses were carried out. *P* < 0.05 was deemed statistically essential.

## Results

### ECHS1 expression levels are higher in GC tumor tissues

**Figure 1:**
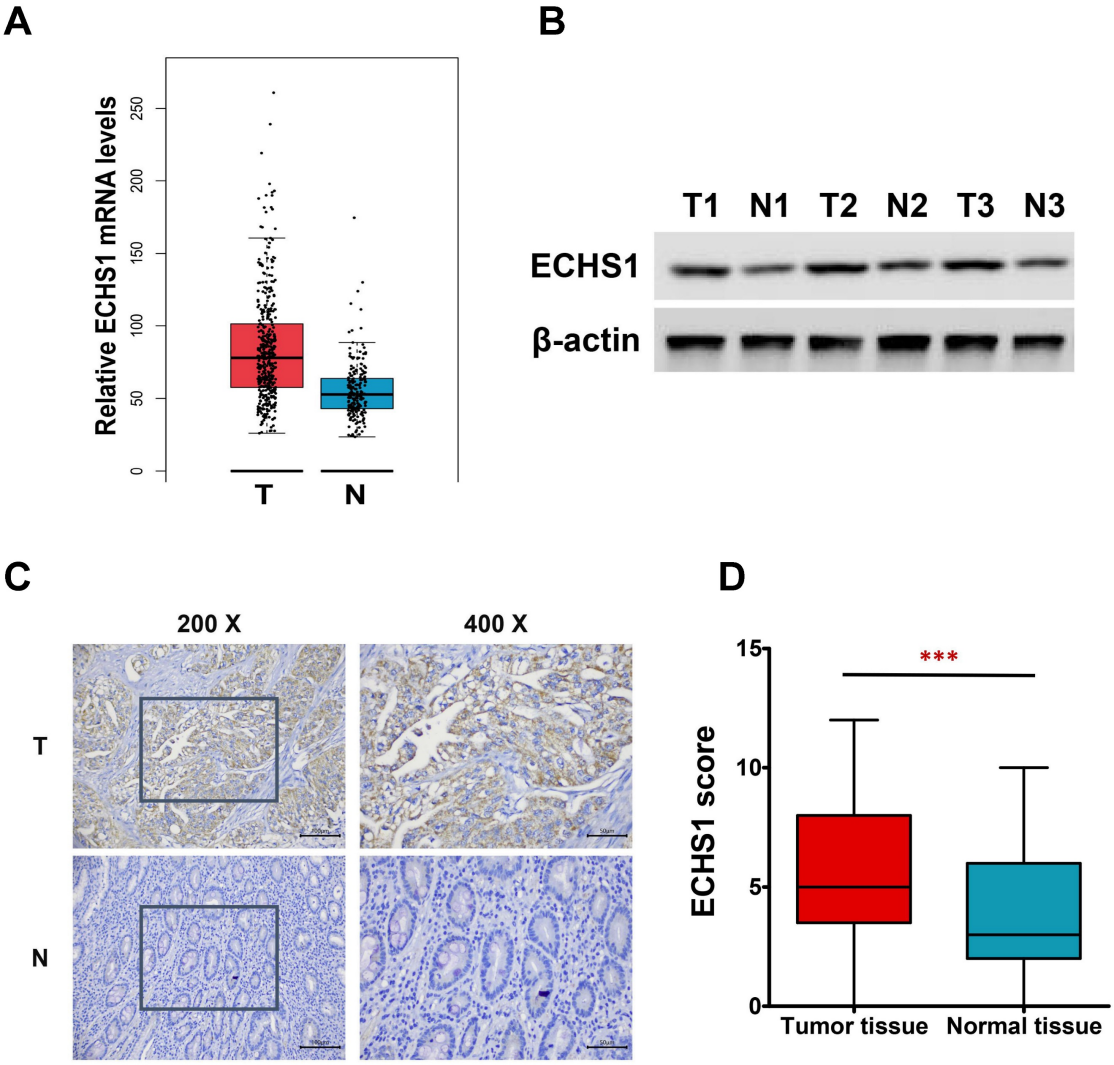
ECHS1 expression in human GC tissues. (A) Relative ECHS1 mRNA expression in stomach adenocarcinoma (STAD) in the TCGA datasheet from the GEPIA. (B) Western blot analysis of ECHS1 in human GC tissues and matched peri-tumoral tissues (n = 3). (C) Representative IHC staining of ECHS1 in human GC tissues and matched peri-tumoral tissues. (D) Analysis of ECHS1 IHC scores in human GC tissues and matched peri-tumoral tissues. ***P < 0.001.

In this investigation, first, the levels of ECHS1 mRNA expression in stomach adenocarcinoma (STAD) were analyzed *via* the GEPIA platform, which revealed increased levels in GC tumor tissues than in healthy tissues (Figure 1A). Next, ECHS1 protein levels in GC and matched peri-tumoral tissues were elucidated by Western blot and IHC analyses, which indicated that the protein expression of ECHS1 was markedly increased in GC tissues than in paired adjacent healthy tissues (Figure 1B-D). Furthermore, IHC also revealed that 42.86% (33/77) GC tissue samples had enhanced staining of ECHS1, whereas high ECHS1 signals were observed in 62.34% (48/77) of tumor-adjacent specimens, which further confirmed that ECHS1 was upregulated in GC tumor tissues.

### Association of ECHS1 expression with clinicopathological characteristics of GC patients

**Figure 2:**
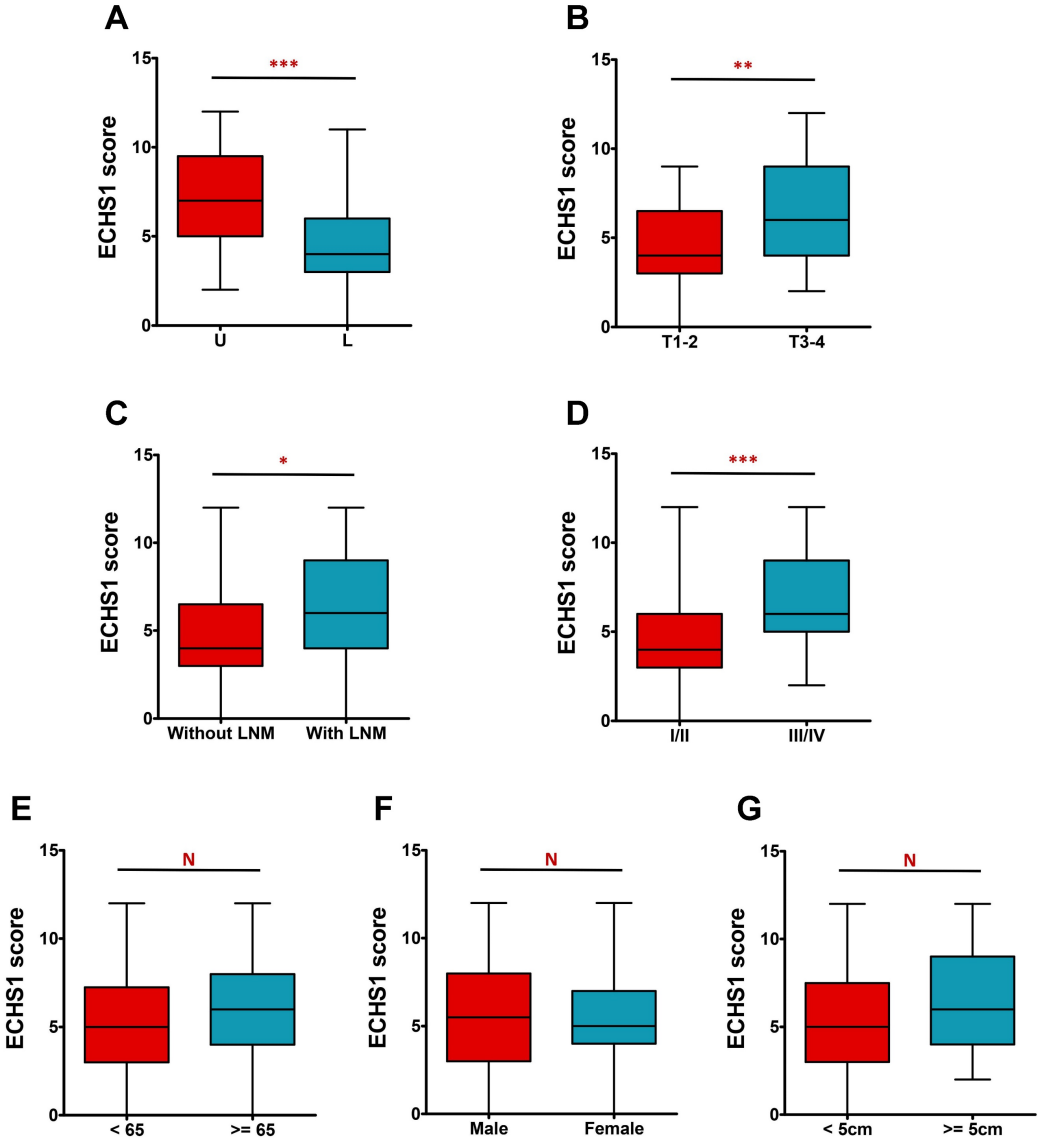
ECHS1 expression in different subgroup of human GC tumor tissues. (A) IHC scores of ECHS1 in GC tumors of different location (U: Upper and middle; L: Lower). (B) IHC scores of ECHS1 in GC tumors with depth of invasion T1-2 or T3-4. (C) IHC scores of ECHS1 in GC tumors with or without LNM. (D) IHC scores of ECHS1 in GC tumors with TNM stage I-II or stage III-IV. (E) IHC scores of ECHS1 in GC tumors with age < 65 or >= 65 years. (F) IHC scores of ECHS1 in GC tumors of of different gender. (G) IHC scores of ECHS1 in GC tumors of different tumor size. N, nonsignificant; *P < 0.05; **P < 0.01; ***P < 0.001.

The IHC analysis further revealed that ECHS1 expression was higher in tumors in the upper and middle parts than in the lower part (*P* < 0.001, Figure 2A). Additionally, deeply invaded GC tumor tissues (T3-4), lymph node metastasis (LNM), and advanced tumor-node-metastasis (TNM) stage (III-IV) indicated enhanced ECHS1 levels than in T1-2 (*P* < 0.01, Figure 2B), without LNM (*P* < 0.05, Figure 2C), and with TNM stage I-II (*P* < 0.001, Figure 2D), respectively. However, ECHS1 levels were not substantially linked with age, gender, or tumor size (*P* > 0.05, Figure 2E-G).

Furthermore, the correlation of ECHS1 expression with clinicopathological characteristics of GC individuals was evaluated. It was indicated that increased ECHS1 expression was markedly linked with tumor location (*P* = 0.002), tumor invasion depth (*P* = 0.007), LNM (*P* = 0.021), and TNM stage (*P* = 0.001) (Table 1). However, ECHS1 levels were not substantially linked with age, tumor size, or gender (*P* > 0.05; Table 1).

### A high expression level of ECHS1 in GC is correlated with poor survival

**Figure 3:**
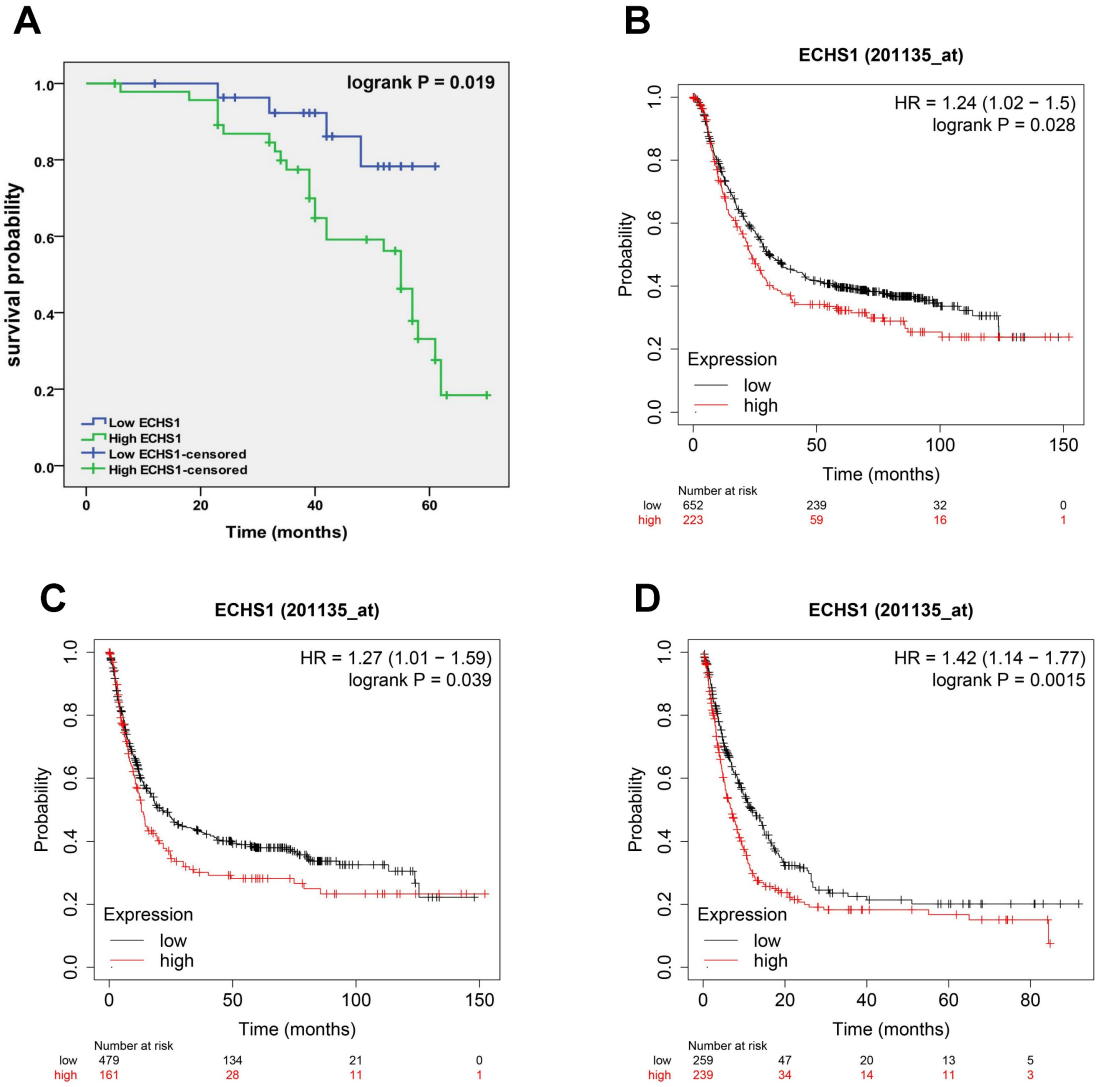
A high expression level of ECHS1 in GC is correlated with poor survival. (A) Overal survival (OS) of GC patients demarcated by ECHS1 expression levels detected by IHC. (B-D) Kaplan-Meier curve for the OS (B), first progression (FP, C) and post progression survival (PPS, D) of patients with GC from Kaplan-Meier plotter databases.

To elucidate the correlation of ECHS1 protein expression with GC patient's prognosis, K-M analysis was carried out, which revealed that the high-expression cohort had shorter OS than the low-expression cohort (Log-rank test, *P* = 0.019, Figure 3A), suggesting that high ECHS1 expression indicated poor GC patient survival outcomes. Furthermore, K-M curves of PPS, FP, and OS for GC patients were acquired from K-M plotter databases and indicated that increased ECHS1 mRNA expression was linked with a shorter OS (Figure 3B, HR = 1.24, *P* = 0.028), FP (Figure 3C, HR = 1.27, *P* = 0.039) and PPS (Figure 3D, HR = 1.42, *P* = 0.0015), consistent with the data acquired from IHC assay. Altogether, these results suggest the prognostic value of ECHS1 in GC.

### ECHS1 expression in different subgroups correlates with the OS of patients with GC

**Figure 4:**
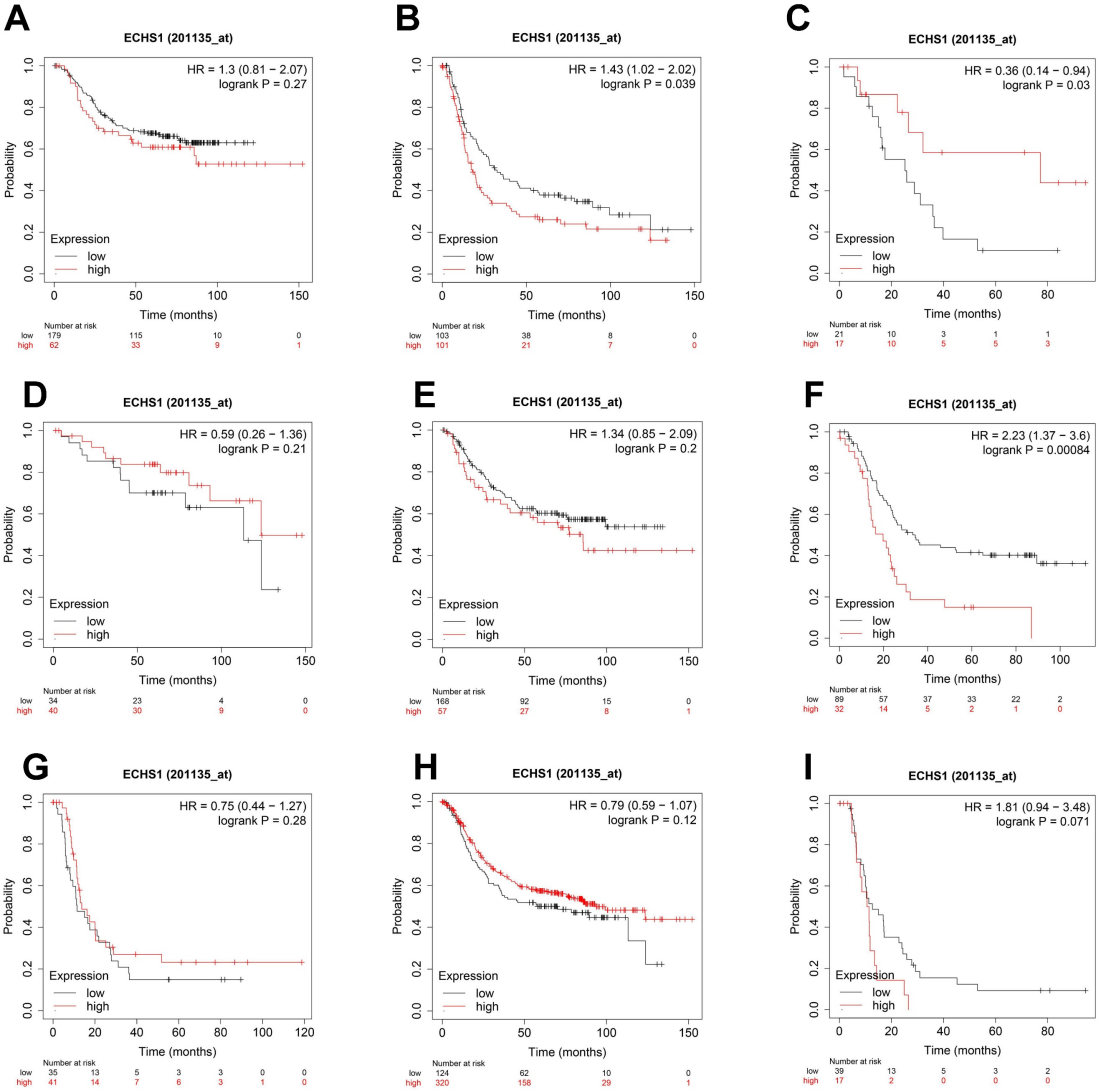
ECHS1 expression in different subgroup correlates with OS of patients with GC from Kaplan-Meier plotter databases. (A-C) Kaplan-Meier survival curve analysis shows OS of GC patients with different depth of invasion (T2 (A), T3 (B), T4 (C)) based on low or high ECHS1 expression. (D-G) Kaplan-Meier survival curve analysis shows OS of GC patients with different N stages (N0 (D), N1 (E), N2 (F), N3 (G)). (H-I) Kaplan-Meier survival curve analysis shows OS of GC patients with different M stages (M0 (H), M1 (I)).

To elucidate the association of ECHS1 mRNA expression in different subgroups and OS, the K-M survival curve analysis from the K-M Plotter database was carried out. According to the subgroup analysis results, OS was markedly shorter in high ECHS1 expressing GC patients than low ECHS1 expressing individuals with T3 stage tumors (Figure 4B, HR = 1.43, *P* = 0.039). However, no substantial relationship existed between the ECHS1 mRNA expression and the OS of T2 stage GC patients (Figure 4A, HR = 1.3, *P* = 0.27). Furthermore, it was revealed that OS was unexpectedly prolonged in high ECHS1 expressing T4 stage GC patients (Figure 4C, HR = 0.36, *P* = 0.03). Considering the N stage, OS was markedly and remarkably reduced in high ECHS1 expressing GC patients than low ECHS1 expressing individuals in the N2 subgroup (Figure 4F, HR = 2.23, *P* = 0.00084). However, no marked association was observed between the ECHS1 mRNA expression and OS of N0 stage GC patients (Figure 4D, HR = 0.59, *P* = 0.21), N1 stage (Figure 4E, HR = 1.34, *P* = 0.2) or N3 stage (Figure 4G, HR = 0.75, *P* = 0.28). Moreover, markedly prolonged OS was observed in high ECHS1 expressing GC patients from the M0 subgroup, with no statistical difference (Figure 4H, HR = 0.79, *P* = 0.12). In the M1 subgroup, OS was shorter in GC patients with high ECHS1 expression (Figure 4I, HR = 1.81, *P* = 0.071, not significant, but marginal).

**Figure 5:**
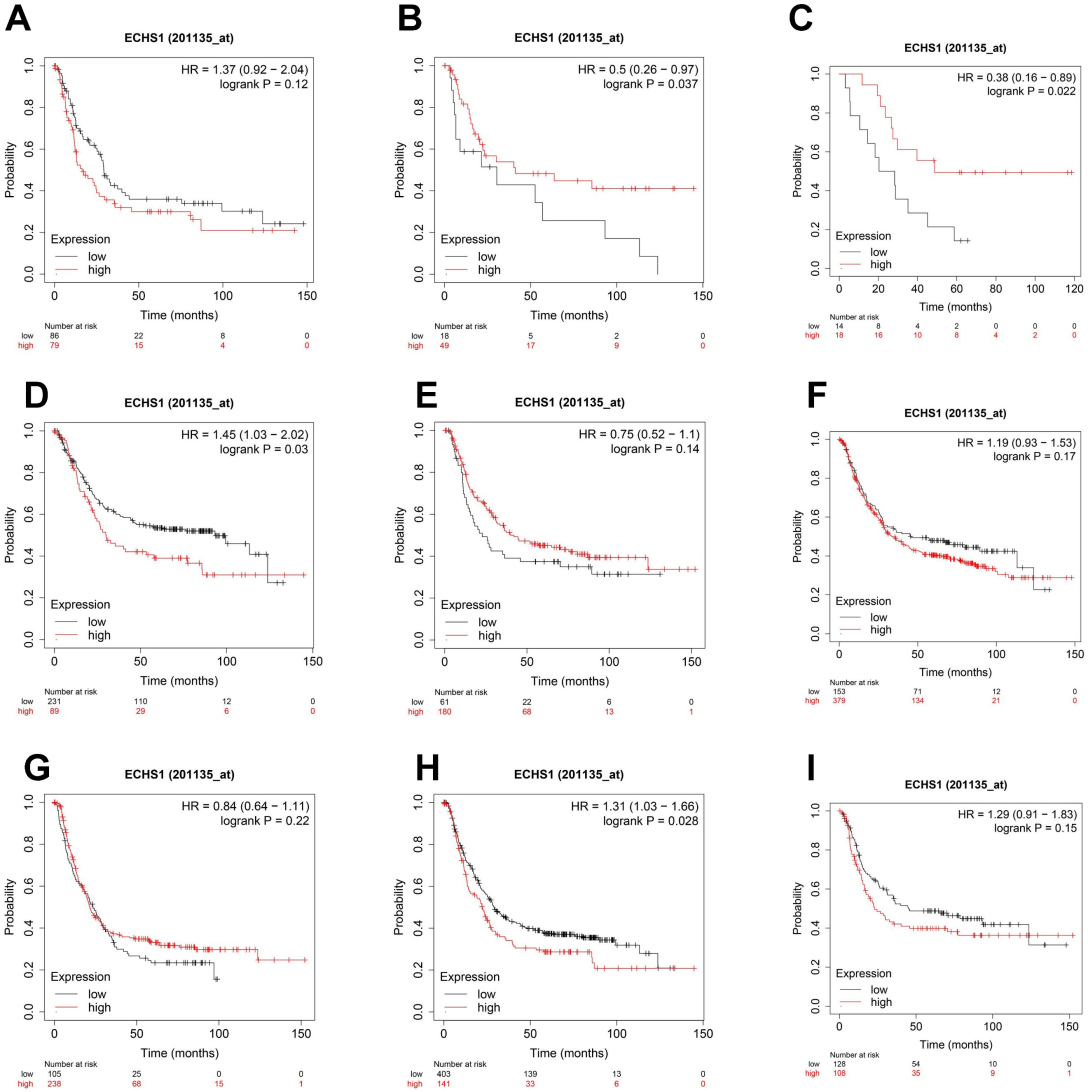
ECHS1 expression in different subgroup correlates with OS of patients with GC from Kaplan-Meier plotter databases. (A-C) Kaplan-Meier survival curve analysis shows OS of GC patients with different degree of differentiation (poor (A), moderate (B), well (C)) based on low or high ECHS1 expression. (D-E) Kaplan-Meier survival curve analysis shows OS of GC patients with different Lauren classification (instestinal (D), diffuse (E)). (F-G) Kaplan-Meier survival curve analysis shows OS of GC patients with different HER2 status (negative (F), positive (G)). (H-I) Kaplan-Meier survival curve analysis shows OS of GC patients with different gender status (male (H), female(I)).

Considering the degree of differentiation, shorter OS was observed in the high ECHS1 expression cohort than in the low ECHS1 expression GC cohort with poor differentiation (Figure 5A, HR = 1.37, *P* = 0.12, not significant, but marginal). Unexpectedly, prolonged OS was indicated in the high ECHS1 expression GC cohort with moderate (Figure 5B, HR = 0.5, *P* = 0.037) and well differentiation. (Figure 5C, HR = 0.38, *P* = 0.022). Based on the intestinal Lauren classification, OS was shorter in the high ECHS1 expression GC cohort than in the low ECHS1 expression cohort (Figure 5D, HR = 1.45, *P* = 0.03). However, the diffuse Lauren classification revealed no notable relationship between the ECHS1 mRNA expression and OS of GC patients (Figure 5E, HR = 0.75, *P* = 0.14). According to the HER2 status, OS was shorter in the high ECHS1 expression GC cohort in the negative group (Figure 5F, HR = 1.19, *P* = 0.17, not significant, but marginal) while an opposite trend was observed in the positive group (Figure 5G, HR = 0.84, *P* = 0.22). Moreover, OS was shorter in the high ECHS1 expression GC cohort for both men (Figure 5H, HR = 1.31, *P* = 0.028) and women (Figure 5I, HR = 1.29, *P* = 0.15, not significant, but marginal).

### ECHS1 promotes GC cell proliferation and migration *in vitro*

**Figure 6:**
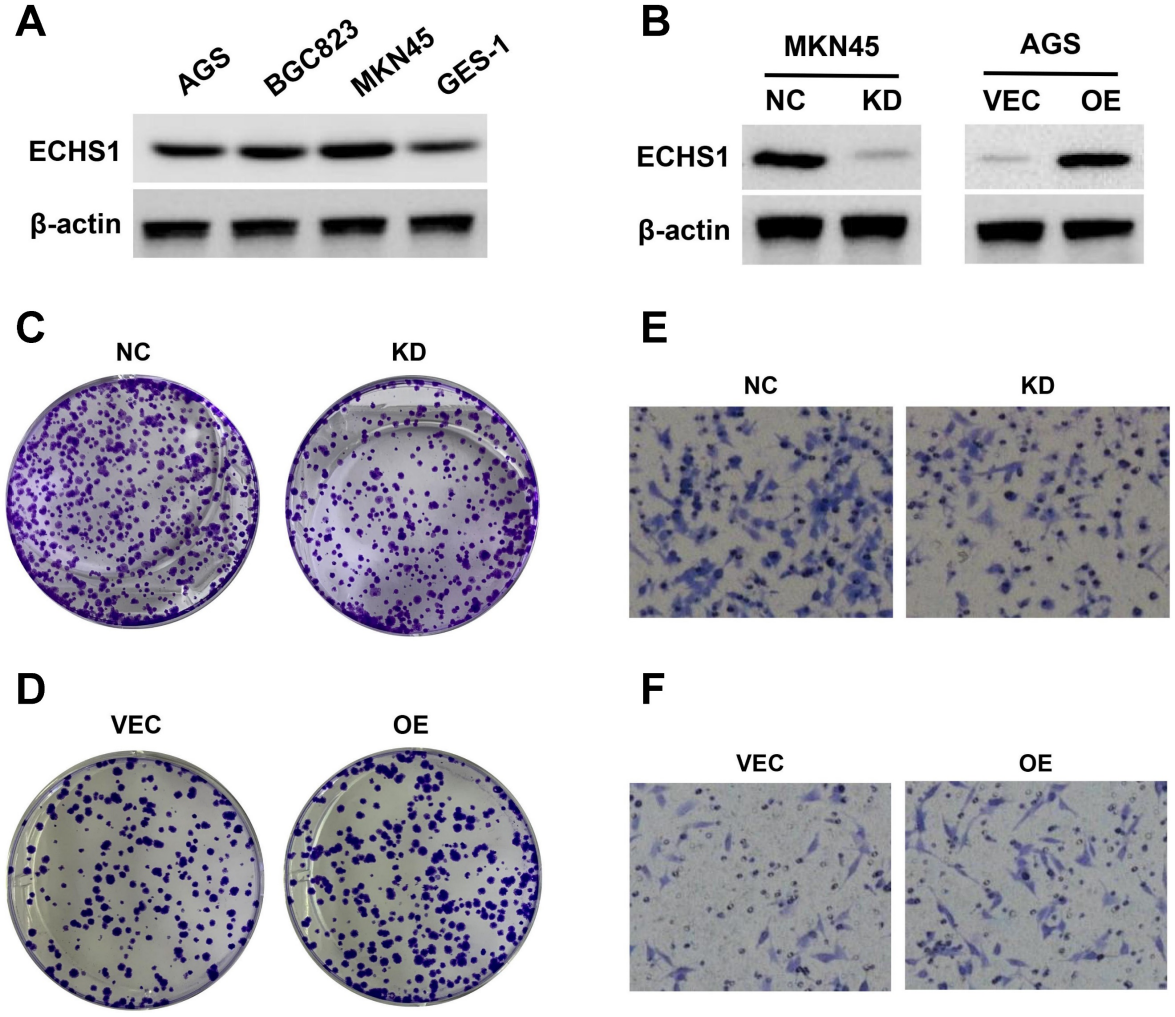
ECHS1 promotes GC cell proliferation and migration in vitro. (A) The ECHS1 protein level in three GC cell lines (AGS, BGC823 and MKN45) and the human gastric mucosal epithelial cells GES-1. (B) The protein expression of ECHS1 in GC cells stably transfected with control-shRNA (NC) or shRNA against ECHS1 (KD) were tested by Western blot (left); The protein expression of ECHS1 in GC cells stably transfected with empty vector (VEC) or plasmid encoding human ECHS1 (OE) were tested by Western blot (right). (C) Colony formation assays were performed in MKN45 cells (NC and KD) and representative photographs are presented. (D) Colony formation assays were performed in AGS cells (VEC and OE) and representative photographs are presented. (E) Migration assays were conducted in MKN45 NC and KD cells and representative photographs are presented. (F) Migration assays were conducted in AGS VEC and OE cells and representative photographs are presented.

Cytological experiments indicated that there was higher ECHS1 expression in GC cell lines (AGS, MKN45, and BGC823) than in normal gastric epithelium (GES-1) cells (Figure 6A). Next, ECHS1 was KD in MKN45 cells, and stable transfectants were generated. The Western blot analysis revealed reduced ECHS1 expression in KD cells than in the controls (NC; Figure 6B). Similarly, plasmids encoding human ECHS1 or empty vector were introduced into AGS cells, and the transfection efficiency was elucidated by Western blotting, which indicated increased ECHS1 expression in plasmids encoding human ECHS1 transfected cells than in empty vector cells (Figure 6B).

The ECHS1 influence on GC cell proliferation was assessed via the colony formation assay. ECHS1-KD suppressed the proliferative ability of MKN45 cells (Figure 6C). Conversely, ECHS1-OE promoted the proliferative ability of AGS cells (Figure 6D). Additionally, the transwell cell migration assay elucidated GC cell's migrative ability, which indicated that the number of membrane penetrating cells in the ECHS1-KD cells was reduced than in the controls (Figure 6E). On the contrary, the migrative ability of AGS cells was enhanced in ECHS1-OE cells (Figure 6F). Suggesting that ECHS1 stimulated the GC cell's ability to proliferate and migrate, confirming the oncogenic effect of ECHS1 on GC cells.

## Discussion

As one of the most frequent malignant tumors and the 3^rd^ primary cause of death by cancer globally, GC poses a great threat to public health 1, 2. Although many advancements have been achieved in current treatment avenues for GC, including surgical procedures, systemic chemotherapy, targeted treatments, and immunotherapies, the long-term prognosis remains substandard. Therefore, identifying novel treatment targets to improve GC patients' prognosis, survival, and therapy response is imperative.

Reprogramming of metabolism is a cancer hallmark; therefore, targeting metabolism is an efficient therapeutic avenue 6. Aberrant metabolism is essential for uncontrolled cellular proliferation, migration, and metastasis to distant tissues 6, 18. The Warburg effect is the earliest described cancer metabolism alteration, whereby normal cells generate energy mainly via oxidative phosphorylation in mitochondria, and cancer cells do so via glycolytic pathway even in the aerobic state 5, 19. However, drugs that target aerobic glycolysis remain unsuccessful clinically due to their limited efficacy and adverse influence 20, 21. With research advancement, it was identified that lipid metabolism is often markedly altered in malignant phenotypic transforming cells to provide energy for the synthesis of the plasma membrane and rapid proliferation 22. On this basis, reprogramming lipid metabolism is a primary cancer metabolism. Currently, reprogrammed lipid metabolism targeting small molecule inhibitors, including SB-204990 (ATP-citrate lyase) and TVB-2640 (FASN), have indicated promising results 23, 24, and some are undergoing clinical trials. Nevertheless, lipid metabolism reprogramming targets, especially those targeting FAO, are still underdeveloped.

ECHS1 is a critical FAO enzyme essentially involved in fatty acid metabolic reprogramming 25. Additionally, ECHS1 is linked with the development of different tumors and induces cancer malignancy by lipid metabolism remodeling and intercellular oncogenic signaling pathways modulation. Furthermore, it has been indicated that ECHS1 can be utilized for early diagnosis and prognosis, suggesting that it could be a potential index for early diagnosis and cancer prognostic. For instance, in colorectal cancer 7, 8, hepatocellular carcinoma 9, 10, and breast cancer 11, ECHS1 expression is markedly upregulated and promotes tumor cells' ability to proliferate, migrate, and chemotherapy resistance. However, in renal cell carcinoma 12, 13, ECHS1 expression is substantially downregulated and could inhibit tumor cell growth. These results suggest that ECHS1 has pro- or anti-cancer properties in pan-cancer.

Research on ECHS1 in GC is rarely reported. Zhu et al. 14 found that ECHS1-KD could inhibit GC cells' ability to proliferate and migrate. Another study 15 revealed elevated ECHS1 expression in GC tissues and that it might be used as a novel marker to assess patient prognosis. This investigation further confirmed the upregulation of mRNA and protein ECHS1 levels in GC tumor tissues. Furthermore, it was observed that increased ECHS1 was markedly linked with GC TNM stage, tumor location, tumor invasion depth, and LNM. Moreover, high ECHS1 expression was also linked with a shorter PPS, FP, and OS, indicating its prognostic value in GC. Furthermore, subgroup analysis suggested that OS was significantly shorter in GC patients with high ECHS1 expression compared to those with low ECHS1 expression belonging to tumors with T3 stage, N2 stage or in instestinal Lauren subgroup. These results indicate the clinical prognostic significance of ECHS1 in GC and that it might be a promising therapeutic target for GC. In addition, cytological experiments showed higher ECHS1 expression in GC cell lines than in the GES-1 cells and promoted GC cells' ability to proliferate and migrate *in vitro*.

There were certain limitations in the present study. First, the function of ECHS1 on GC tumorigenesis was not detected *in vivo*. Second, multi-level suppression of ECHS1 in GC cells was not performed to further confirm the findings. Third, the underlying mechanism of ECHS1 oncogenic roles in GC was not investigated and further research should be undertaken in the future study.

In conclusion, this investigation indicated upregulation of ECHS1 in GC tumor tissues, which was associated with tumor location, TNM stage, tumor invasion depth, LNM, and poor survivals. Cytological experiments showed that ECHS1 promoted GC cells' ability to proliferate and migrate *in vitro*. Therefore, this investigation highlights ECHS1 as a novel target for treating GC.

## Figures and Tables

**Table 1:** Correlation between ECHS1 protein expression and clinicopathological parameters of 77 GC patients.

Variables	Number	ECHS1		χ^2^	*P* value
		None or low	High		
Age (years)					
<65	38	15 (39.5%)	23 (60.5%)	0.105	0.746
>=65	39	14 (35.9%)	25 (64.1%)		
Gender					
Male	52	20 (38.5%)	32 (61.5%)	0.044	0.835
Female	25	9 (36.0%)	16 (64.0%)		
Tumor size					
<5cm	37	16 (43.2%)	21 (56.8%)	0.945	0.331
>=5cm	40	13 (32.5%)	27 (67.5%)		
Tumor location					
U	41	9 (22.0%)	32 (78.0%)	9.220	0.002**
L	36	20 (55.6%)	16 (44.4%)		
Depth of invasion					
T1-2	21	13 (61.9%)	8 (38.1%)	7.228	0.007**
T3-4	56	16 (28.6%)	40 (71.4%)		
Lymph node metastasis					
No	25	14 (56.0%)	11 (44.0%)	5.302	0.021*
Yes	52	15 (28.8%)	37 (71.2%)		
TNM Stage					
I/II	29	18 (62.1%)	11 (37.9%)	11.803	0.001**
III/IV	48	11 (22.9%)	37 (77.1%)		

*P < 0.05; **P < 0.01
